# Prognostic factors for cranial deformities in infancy: a retrospective cohort study

**DOI:** 10.3389/fped.2026.1822648

**Published:** 2026-06-25

**Authors:** Rui Li, Qiong Jia, Jing Wang, Xinyu Huang, Jing Yu, Jinyang Bai, Hua Zhang

**Affiliations:** 1 Department of Pediatrics, Peking University Third Hospital, Beijing, China; 2 Department of Pediatrics, People’s Hospital of Qiandongnan Miao and Dong Autonomous Prefecture, Kaili, Guizhou, China; 3 Clinical Epidemiology Research Center, Peking University Third Hospital, Beijing, China

**Keywords:** positional cranial deformity, age at diagnosis, severity of deformity, recovery time, infant

## Abstract

**Purpose:**

This study aimed to investigate the prognostic factors on the natural recovery time of positional cranial deformities in infants.

**Methods:**

A retrospective cohort study was conducted including 1,219 infants diagnosed with positional cranial deformities who attended the Children's Health Development Center of our hospital on at least two occasions between January 2022 and April 2025. The cohort comprised 228 preterm infants and 991 full-term infants. Cranial parameters, including cranial vault asymmetry (CVA), cranial vault asymmetry index (CVAI), and cephalic ratio (CR), were measured using the STARscanner 2.0 system. Median recovery time was estimated using survival analysis. The effects of age, sex, gestational age at birth, maternal age, birth weight, cranial shape, and severity of deformity on recovery time were evaluated using multivariate proportional hazards Cox regression analyses.

**Results:**

Multivariate analysis demonstrated that age was an independent predictor of natural recovery (hazard ratio = 0.44, *P* < 0.001), with younger age exhibiting a significantly faster recovery. Deformity severity was a key determinant, as infants with mild deformities recovering significantly faster than those with severe deformities (*P* < 0.001). In contrast, sex and cranial shape were not significantly associated with recovery time.

**Conclusion:**

Younger age at diagnosis and milder deformity severity were associated with a higher likelihood and faster rate of natural recovery from positional cranial deformities in infants. Enhanced follow-up and targeted management are recommended for infants diagnosed at an older age at diagnosis and for those presenting with more severe deformities in clinical practice.

## Highlight

- Infants of younger age at diagnosis and with milder cranial deformities had a greater likelihood and faster rate of natural recovery of positional cranial deformities.
- Strengthening of follow-up and management for infants of older age at diagnosis and with deformities of higher severity is recommended in clinical practice.

## 1 Introduction

Infantile positional cranial deformity, characterized by asymmetrical head shape in infants, is manifested as differences in the height and width of the head or skull, and may be accompanied by abnormalities in facial structures, such as the ears or eyes (1). Studies indicate that the overall incidence of cranial deformities in infants is approximately 13% (2). Recently, the American Academy of Pediatrics has recommended that parents adopt the “supine sleeping position” for their infants (3). With the promotion of the “supine sleeping position” campaign, the incidence of cranial deformities reported in the literature increased to 20%–46%, with the rate among preterm infants reaching 54.0–77.2% (4–6). In this study, the overall incidence of infantile positional cranial deformities was 56.9%, with rates of 76.8% and 54.96% observed in preterm and full-term infants, respectively. Cranial deformities have become a common health issue in infant development. Besides affecting an infant's appearance and leading to facial asymmetry, they may impact social interactions and psychological development or affect their gross motor development. Certain infants with severe cranial deformities may suffer comorbid cognitive impairment (5–8). Therefore, this issue has attracted attention in the field of infant growth and development. Although existing research suggests that mild cranial deformities resolve naturally (9–12), there are few reports on the key factors influencing the natural recovery process, particularly the relationships of age at diagnosis, deformity type, and severity with recovery time.

Therefore, this retrospective cohort study design used survival analysis to explore the natural recovery process of infants with positional cranial deformities of of different ages at diagnosis, types, and severity levels, providing individualized clinical guidance for the early identification of high-risk infants requiring intervention.

## 2 Participants and methods

### 2.1 Participants

The participants were infants who underwent cranial monitoring at the Children's Health Development Center of our hospital between January 1, 2022 and April 30, 2025. The inclusion criteria were as follows; (1) infants aged at diagnosis 0–6 months upon initial cranial measurement, (2) diagnosed with positional cranial deformity through standardized assessment, (3) infants with at least two consecutive measurement data points, and (4) consent for study participation by a family member. Exclusion criteria were as follows; (1) comorbid pathological cranial abnormalities, such as craniosynostosis or other congenital craniofacial syndromes, (2) missing data, and (3) infants who underwent cranial correction interventions such as helmet or surgery during the study period. This study was approved by the Medical Research Ethics Committee of our hospital (Approval Number: IRB00006761-M20250752).

### 2.2 Measurement methods for cranial deformities

**Figure 1:**
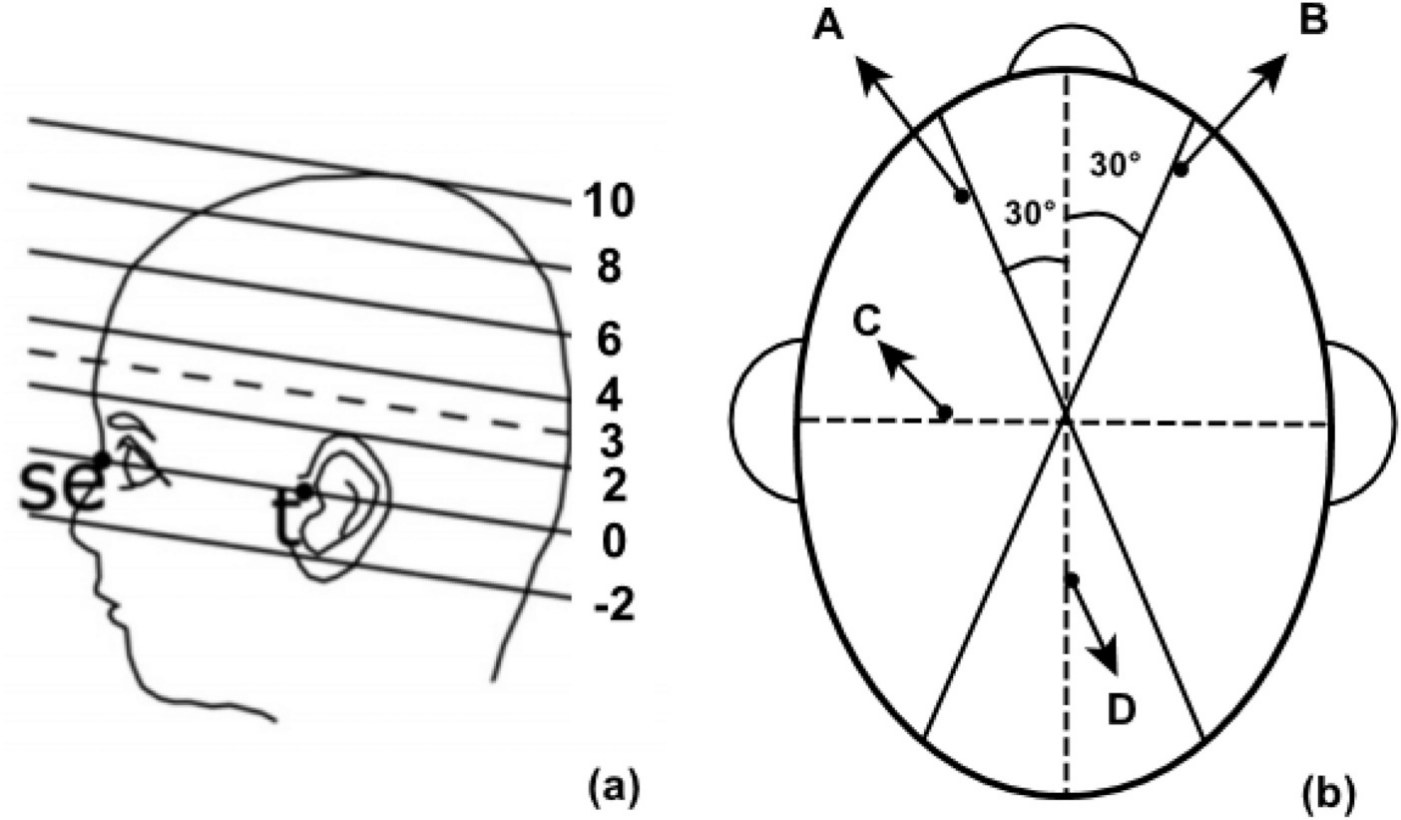
Cranial Morphology Scan Images: **(a)** schematic illustration of the plane corresponding to level 3; **(b)** schematic illustration of measurement indicators (13).

Three-dimensional (3D) cranial scanning was performed using the STARscanner 2.0 laser system, and 3D images were captured by processing the cranial data with specialized software. The horizontal reference plane (level 0) was defined by the glabella (midpoint between the eyes) and tragus of the left and right ears. Subsequently, the region from level 0 to the top of the head was divided into 10 equal segments from bottom to top. All study parameters were derived from level 3 cross-sections (13) (Figure 1a).

On the level 3 cross-section, the vertical distances between the anterior and posterior aspects of the cross-section at 30° to the left and right of the midline were measured and designated as A and B, respectively (A > B). Cranial vault asymmetry (CVA) = A-B. Cranial vault asymmetry index (CVAI) = (A-B)/A × 100%. The width and length of the midline of the level 3 cross-section were denoted as C and D, respectively. Cephalic ratio (CR) = C/D (Figure 1b) (13).

### 2.3 Diagnostic criteria for cranial deformities

Based on international diagnostic criteria, previous literature, and clinical experience, according to value of CVA, CVAI and CR, all cases can be divided into three level, mild, moderate and severe. This study adopted the following diagnostic criteria for positional cranial:

(1) Plagiocephaly: Uneven distribution of pressure on both sides of the skull, causing unilateral cranial flattening and an increased diagonal difference of the skull. The threshold for abnormal values was set as CVA ≥ 3 mm or CVAI ≥ 3.5% (13–15). Severity was defined as mild (CVA 3–10 mm or CVAI 3.5%–6.25%), moderate (CVA 10–12 mm or CVAI 6.25%–8.75%), and severe (CVA > 12 mm or CVAI 8.75–11.0%).
(2) Brachycephaly (flat-head deformity): An abnormal shortening of the skull that results in an excessively wide head with an increased head width/head length ratio. The threshold for abnormal values was set as CR ≥ 82%. Severity was classified as mild (CR 82%–90%), moderate (90%–100%), and severe (>100%).
(3) Asymmetric brachycephaly: Combined brachycephaly and plagiocephaly. The threshold for abnormal values was set as CR ≥ 82%, combined with CVA ≥ 3 mm or CVAI ≥ 3.5%. Severity was determined by the greater severity level between brachycephaly and plagiocephaly.
(4) Scaphocephaly: The anteroposterior diameter is significantly greater than the transverse diameter, resulting in a long and narrow head shape. The threshold for abnormal values was set as CR < 74%. Severity was defined as mild (CR 74%–76%), moderate (70%–74%), and severe (<70%).

### 2.4 Data collection

This study was a retrospective cohort study, and the sample size was estimated based on pre-trial data. With α set at 0.05, β at 0.20 (80% statistical power), and an expected hazard ratio (HR) for correction time between preterm and full-term infants of 1.8, the minimum required sample size was calculated as 120 cases using PASS 15.0 software. Considering a 10% loss-to-follow-up rate, it was planned to include at least 132 children. Ultimately, this study included 1,219 children, meeting the sample size requirement.

All enrolled children received regular outpatient follow-up, with visits scheduled every 3 months from the initial diagnosis until deformity correction or until the child reached 18 months of age. During each follow-up, physical examination findings were recorded, and cranial deformity improvement was assessed using the STARscanner 2.0 system. The outcome measure was “time to deformity correction”, defined as the interval from the initial diagnosis to the clinical evaluation confirming correction of the deformity.

All clinical data were obtained from patient records. Demographic characteristics, including sex, gestational age at birth, birth weight, and maternal age, were analyzed. Clinical characteristics used for analysis included the age at initial diagnosis of cranial deformity, type of cranial deformity, and severity of the deformity.

### 2.5 Statistical methods

Statistical analysis was performed using SPSS 27.0 statistical software. Normally distributed data were expressed as `mean ± standard deviation, non-normally distributed data as median (interquartile range), and count data as number of cases (percentage). Intergroup comparisons were performed using the *t*-test, Mann–Whitney *U*-test, Kruskal–Wallis *H*-test, or Chi-square test based on data type and distribution characteristics. Survival analysis was employed to examine the independent effects of age at diagnosis on recovery time in preterm and full-tem infants. Statistical significance was set at *P* < 0.05. Kaplan–Meier survival curves were plotted to illustrate the time to deformity correction for both preterm and full-term infants, with the Log-rank test used to compare differences between the two groups. Additionally, a multivariate Cox proportional hazards regression model was established to investigate the independent effect of diagnostic age (in months) on the recovery time in both preterm and term infants.

## 3 Results

### 3.1 Demographic characteristics

**Figure 2:**
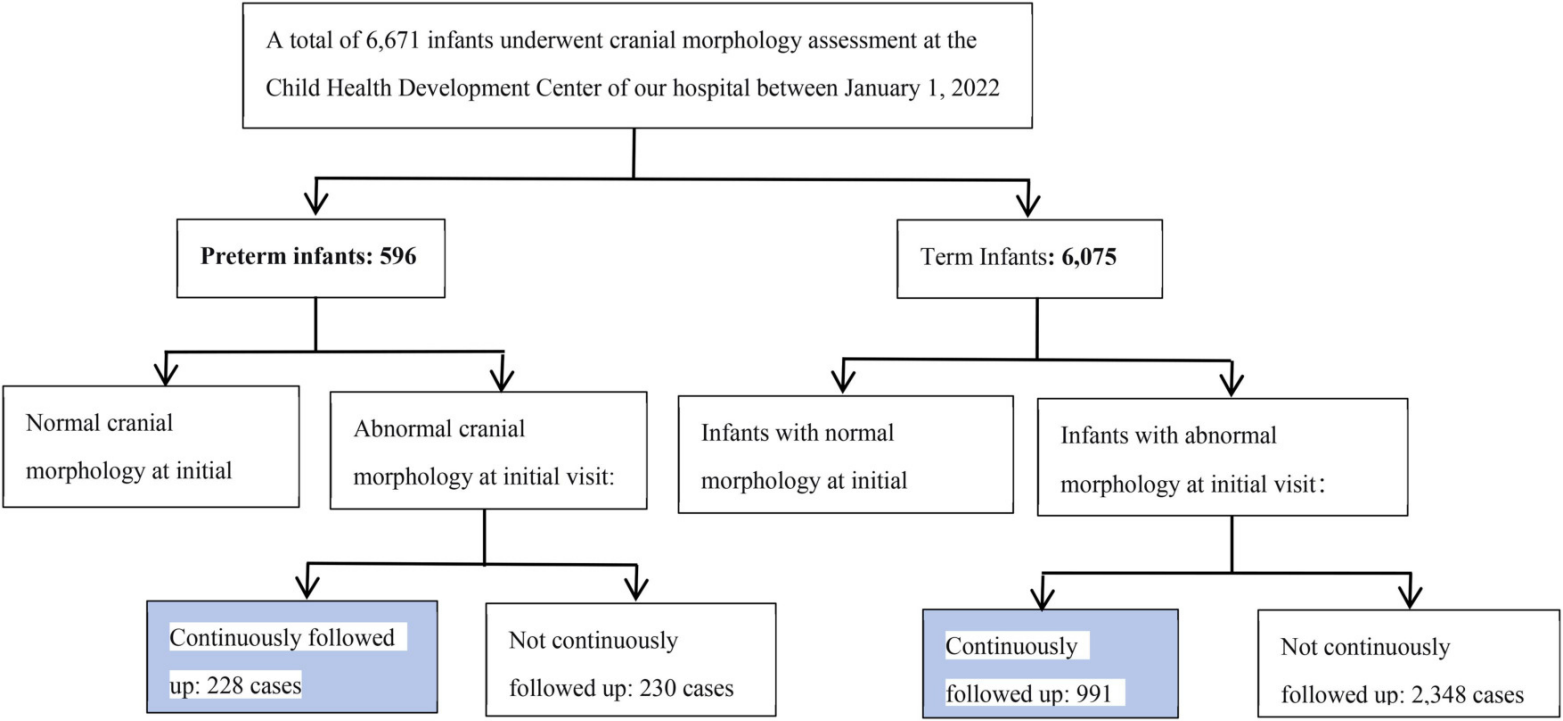
Flowchart of study participant enrollment.

Between January 1, 2022 and April 30, 2025, 6,671 infants underwent cranial monitoring at the Children's Health Development Center of our hospital. Participant inclusion process is shown in Figure 2. After excluding 2,874 infants with normal cranial shape at their first visit and 2,578 infants without continuous follow-up, this study included 1,219 infants, among which, 228 were preterm and 991 were full-term infants [636 boys (52.2%), 583 girls (47.8%)]. Participants were grouped based on the corrected age at initial visit; 0–2 months (170 cases, 13.9%), 3–4 months (871 cases, 71.5%), and 5–6 months (178 cases, 14.6%).

Comparison of baseline characteristics among infants of the different age groups are shown in Table 1. Results indicated that the 0–2-month group had significantly lower gestational age at diagnosis at birth and birth weight and greater severity of cranial deformities compared to the 3–4- and 5–6-month groups (*P* < 0.001). There were no significant differences in sex, maternal age, or cranial shape distribution among the three groups (*P* > 0.05).

**Table 1:** General baseline characteristics.

Variables	Overall (*n* = 1,219)	0–2 months (*n* = 170)	3–4 months (*n* = 817)	5–6 months (*n* = 178)	H/U	*P*
Sex (*n*, %)					0.021	0.990
Male	636 (52.17)	89 (52.35)	455 (55.69)	92 (51.69)		
Female	583 (47.83)	81 (47.65)	416 (44.31)	86 (48.31)	148.004	**<0** **.** **001**
Gestational Age at Birth (weeks, mean ± SD)	37.65 ± 3.54	33.99 ± 3.49	38.18 ± 3.55	38.51 ± 3.53		
Birth Weight	3,003.78 ± 757.20	2,228.52 ± 954.37	3,117.87 ± 642.04	3,185.88 ± 612.35	127.412	**<0** **.** **001**
Maternal Age	33.77 ± 9.32	33.83 ± 4.17	33.86 ± 10.74	33.26 ± 3.62	3.22	0.200
Cranial Morphology Type					2.750	0.840
Plagiocephaly	707 (58.00)	96 (56.47)	513 (62.79)	98 (55.06)		
Brachycephaly	51 (4.18)	9 (5.29)	36 (4.41)	6 (3.37)		
Asymmetric Brachycephaly	73 (5.99)	12 (7.06)	51 (6.24)	10 (5.62)		
Scaphocephaly	388 (31.83)	53 (31.18)	271 (33.16)	64 (35.95)		
Severity of Abnormality (*n*, %)					19.293	**<0** **.** **001**
Mild	884 (72.52)	101 (59.41)	653 (79.93)	130 (73.03)		
Moderate	232 (19.03)	42 (24.71)	154 (18.84)	36 (20.22)		
Severe	103 (8.45)	27 (15.88)	64 (7.83)	12 (6.74)		

For preterm infants, the 5–6-month group had significantly higher birth weight compared to the other two groups (*P* = 0.001). Differences in gestational age at birth among the three groups were statistically significant (*P* = 0.001). There were no significant differences in the distribution of sex, maternal age, cranial shape, or severity of deformity among the groups (*P* > 0.05) (Supplementary Table S1).

There were no significant differences in all baseline indicators across the age at diagnosis groups among term infants (*P* > 0.05), which indicated comparability among the various age at diagnosis groups of full-term infants (Supplementary Table S2).

### 3.2 Survival analysis of cranial recovery time

#### 3.2.1 Survival analysis of cranial recovery time

In medical research, when the primary observation outcome is the time at which a certain interesting event occurs, survival analysis methods are often employed. This time is generally referred to as survival times (16). In this study, the survival time is the period from the first diagnosis of cranial deformity to the recovery of their cranial shape. Due to the fact that some subjects have not recovered by the end of the study, resulting in censored data, and the recovery time usually shows a skewed distribution, survival analysis methods are required for statistical processing.

#### 3.2.2 Survival analysis of cranial recovery time in infants of different age at diagnosis groups

**Figure 3:**
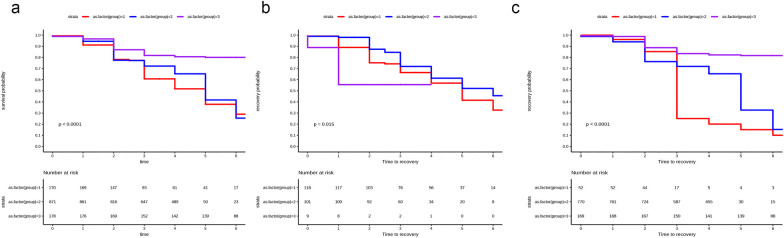
Kaplan–Meier Survival Curves for Cranial Morphology Recovery Time by Diagnostic age group. **(a)** all; **(b)** Preterm Infants; (c) Term Infants. Group1 is diagnostic age 0–2 months, group 2 is diagnostic age 3–4 months, Group 3 is diagnostic age 5–6 months.

Kaplan–Meier survival analysis revealed that the median recovery time was 5.0 months in the 0–2- and the 3–4-month groups, whereas the 5–6-month group had an extended recovery time of 7.0 months (Table 2, Figure 3a). Results indicated that the natural recovery time for cranial deformities was shorter for infants of younger age at diagnosis.

**Table 2:** Survival analysis of cranial morphology recovery time in infants by Diagnostic age group.

Diagnostic age	Recovery time in infants (months)		Recovery time in preterm infants (months)		Recovery time in term infants (months)		*P*
	Median	95% CI	Median	95% CI	Median	95% CI	
0–2 months	5.00	4.16–5.83	5.00	4.16–5.83	3.00	2.69–3.31	0.480
3–4 months	5.00	4.62–5.38	6.00	4.62–5.38	5.00	4.64–5.36	0.480
5–6 months	7.00	–	–	–	7.00	–	–
Overall	7.00	6.22–7.78	5.00	4.20–5.79	7.00	6.59–7.41	1.000

Kaplan–Meier survival curves for cranial recovery across different age at diagnosis groups (Figure 3a) exhibit the dynamic changes in natural recovery rates among infants of different ages at diagnosis. The curves of younger infants exhibited an earlier and more rapid decline. The curve for the 0–2-month group declined rapidly at the earliest stage, indicating that infants in this group had the highest proportion and fastest natural recovery rate within the first few months after birth. For the 3–4-month group, the trend of decrease was similar to that of the 0–2-month group, albeit with a slight delay. The recovery process was concentrated within a relatively short period of time. The curve for the 5–6-month group was the flattest and consistently remained at the top, indicating that infants in this group had the slowest spontaneous recovery rate. The proportion of infants who did not recover remained higher than that of the younger age groups.

#### 3.2.3 Survival analysis of cranial recovery time in preterm infants of different age groups

Kaplan–Meier survival analysis revealed that the median recovery time was 5.0 months in the 0–2-month group. The 3–4-month group exhibited a longer median recovery time of 6.0 months, whereas the median recovery time of the 5–6-month group could not be effectively estimated (Table 2, Figure 3b). Results indicated that preterm infants of younger gestational age had a relatively shorter spontaneous cranial recovery time.

Kaplan–Meier survival curves for cranial recovery time in preterm infants across different age at diagnosis groups is shown in Figure 3b. The curve for the 0–2-month group declined rapidly in the early stage, indicating a higher proportion and rate of spontaneous recovery in the preterm infants during the initial period. In the 3–4-month group, the decline in the curve was relatively gradual and recovery process was slightly delayed. The curve for the 5–6-month age group showed a slower decrease, indicating a longer recovery time.

#### 3.2.4 Survival analysis of cranial recovery time in full-term infants of different age at diagnosis groups

The median recovery time was shortest in the 0–2-month term infant group at 3.0 months, (3–4-month group; 5.0 months, 5–6-month group; 7.0 months) (Table 2, Figure 3c).

Kaplan–Meier survival curves for each age at diagnosis group of full-term infants were largely consistent with those of the overall population. For infants of younger age at diagnosis, the curves showed an earlier and more rapid decline, confirming that age at diagnosis is a key factor influencing the rate of spontaneous recovery.

### 3.3 Cox regression analysis of factors affecting cranial recovery time

Cox regression analyses were separately performed in the overall population, preterm infants, and full-term infants for further exploration of factors influencing recovery time.

Multivariate Cox regression analysis indicated that age at diagnosis group, severity of deformity, and gestational age at birth were independent predictors influencing spontaneous cranial recovery time in infants (*P* < 0.05) (Table 3). Infants of younger age at diagnosis had a faster recovery rate [hazard ratio (HR) = 0.521, 95% confidence interval (CI); 0.424–0.641]. Compared with infants with mild deformities, those with moderate (HR = 1.929, 95% CI：1.457–2.554) and severe deformities (HR = 6.785, 95% CI: 3.359–13.703) exhibited a lower likelihood of recovery. Gestational age at birth was associated with recovery rate (HR = 1.001, 95% CI; 1.000–1.002). Cranial shape, age group, sex, birth weight, and maternal age were not independent influencing factors in multivariate analysis (*P* > 0.05).

**Table 3:** Multivariate cox regression analysis of factors influencing the time to spontaneous recovery of infant cranial morphology.

Variables	Infants			Preterm infants			Term infants		
	*P*	HR	95% CI	*P*	HR	95% CI	*P*	HR	95% CI
Diagnostic Age Group	<0.001	0.521	0.424–0.641	0.015	0.271	0.095–0.777	<0.001	0.444	0.350–0.564
Severity of Abnormality	<0.001			<0.001	0.459	0.330–0.637	<0.001		
Moderate vs. Mild	<0.001	1.929	1.457–2.554	0.022	1.705	1.081–2.687	<0.001	2.410	1.656–3.506
Severe vs. Mild	<0.001	6.785	3.359–13.703	<0.001	4.236	2.021–8.878	0.892	–	–
Gestational Age at Birth	0.015	1.001	1.000–1.002	0.002	1.002	1.001–1.003	0.792	0.998	0.985–1.012
Preterm Birth Status	0.136	0.753	0.518–1.094						
Birth Weight	0.101	1.000	1.000–1.000	0.269	1.000	0.999–1.000	0.046	1.000	1.000–1.000
Cranial Morphology Type	0.428	1.030	0.957–1.109	0.079	1.166	0.983–1.383	0.646	1.020	0.938–1.110
Sex	0.957	1.005	0.832–1.214	0.134	1.371	0.907–2.073	0.349	0.902	0.728–1.119
Maternal Age	0.995	1.000	0.988–1.012	0.686	1.011	0.959–1.066	0.892	0.999	0.984–1.014

Multivariate analysis in preterm infants indicated that age at diagnosis group, severity of deformity, and gestational age at birth were independent predictors influencing cranial recovery in preterm infants (Table 3). Recovery rates for the 0–2- and 3–4-month groups were significantly faster than that of the 5–6-month group (*P* < 0.05). The severity of deformity demonstrated a highly significant independent predictive value (HR = 0.459, *P* < 0.001). Recovery rate showed a decrease of >50% for each level of increase in deformity severity. Gestational age at birth emerged as an independent predictor in the multivariate model (*P* = 0.002), suggesting that higher maturity served as an independent protective factor promoting natural cranial recovery in preterm infants.

Multivariate analysis in full-term infants showed that age group was an independent predictor of cranial recovery rate (HR = 0.444, 95% CI; 0.350–0.564, *P* < 0.001), reaffirming that younger age at diagnosis was a strong protective factor that promoted recovery (Table 3). Birth weight demonstrated independent statistical significance (HR = 1.000, *P* = 0.046). Sex, cranial shape, maternal age, and gestational age at birth were not independent influencing factors.

## 4 Discussion

In this study, a retrospective analysis was conducted in 1,219 infants with positional cranial deformities to investigate the influence of age and severity of deformity on natural recovery process. Results indicated that younger age at diagnosis and milder deformities were associated with faster and a greater likelihood of natural recovery, further validated through multivariate Cox regression analysis. Age at diagnosis and severity of cranial deformity were identified as independent key factors influencing natural recovery of positional cranial deformities in infants. This finding corroborates previous clinical observations (10) and provides evidence-based support through rigorous survival analysis and multivariate Cox regression models. Research has indicated that the natural history of infantile positional cranial deformities is jointly shaped by developmental timing and the severity of initial morphological defects. This lays the foundation for implementing individualized, precision-based clinical management pathways.

### 4.1 Relationship between different ages at diagnosis and recovery time

The study results indicated that age at diagnosis was negatively correlated with the rate of spontaneous recovery (HR = 0.444, *P* < 0.001). Median recovery duration was significantly longer in the 5–6-month group (7.0 months) than in the 0–4-month group (5.0 months). This finding corroborates Hiroshi's view (17) that the skull exhibits high plasticity in early infancy. From a developmental perspective, the cranial sutures have not yet fully closed, and the membranous bone possesses a strong capacity for remodeling to accommodate explosive brain growth. Previous research has indicated that during the first year of life, an infant's brain volume can increase to more than twice its birth size (18). Such rapid brain growth exerts the most sustained, inside-out corrective force on cranial shape. Infants of a younger age possess a greater “molding potential” for brain growth, leading to higher efficiency and likelihood of spontaneous correction of morphological deformities caused by external pressure. As the infant grows older, the skull gradually hardens, and the driving force for spontaneous recovery shows a corresponding decline.

### 4.2 Multivariate analysis of factors affecting recovery time

Multivariate analysis revealed that infants with mild deformities recovered 8.142 times faster than those with severe deformities, highlighting the decisive role of initial severity in prognostic assessment and consistent with Di Chiara (19) and de Sousa (20). Severe cranial deformities are often not merely limited to mild tilting of a single bone plate, but frequently involve complex deformities of the entire cranial vault. These may be accompanied by compensatory frontal or occipital protrusions and result in facial asymmetry (1, 5). Such severe morphological alterations exceed the compensatory potential of normal brain growth, resulting in a morphological “trap”. Therefore, although all positional cranial deformities theoretically possess a tendency toward spontaneous resolution, the spontaneous recovery pathway for severe deformities may be extremely protracted or even incomplete. This carries the risk of leaving permanent cosmetic defects and may even be associated with future neurodevelopmental delays (21–23).

### 4.3 Analysis of differences between preterm and term infants

The subgroup analysis demonstrated the distinctiveness of the preterm infant population. In preterm infants, gestational age at birth emerged as an independent predictor of recovery time (HR = 1.002, *P* = 0.002). This indicates that “in utero maturity” remains a crucial biological marker for predicting postnatal self-correction capacity (24), which reflects the dual challenges faced by cranial development in preterm infants, in which the skulls of preterm infants are softer and more prone to deformation (19) and they experience significant “catch-up growth” after birth. Although this presents an opportunity for cranial correction, it poses a challenge as it is often accompanied by hypotonia and limited mobility, thereby restricting active positional changes (25). Therefore, cranial management for preterm infants requires a more meticulous and proactive approach, considering the corrected age at diagnosis, gestational age at birth, and neuromotor development status of the infants.

### 4.4 Clinical significance and implications

The study findings can offer direct guidance for clinical practice. Traditionally, a “wait-and-see” approach has been adopted for mild cranial deformities. However, this study provides quantitative evidence for risk-stratified management.

Low-risk groups (younger infants or mild deformities) should be prioritized as key targets for health education. By guiding parents to implement standardized position management, such as supervised prone positioning during waking hours, alternating sleeping positions, and encouraging head turning) (26), supplemented by regular monitoring, most infants can achieve satisfactory spontaneous recovery. This approach avoids unnecessary medical interventions and associated family anxiety and financial burden.

For high-risk groups (elder age at diagnosis or moderate-to-severe deformities), closer follow-up should be initiated, and the necessity of active intervention should be discussed as appropriate. For infants with severe deformities with no improvement or slow progress after 1–2 months of standardized positional management, orthotic helmet therapy should be introduced as a key treatment option (27). Our data indicated that for such infants, excessive waiting may result in missing the optimal window for orthopedic treatment between 4 and 8 months of age at diagnosis (9).

### 4.5 Research limitations and future directions

This study has several limitations. First, the single-center retrospective nature of this study implies that selection bias may exist despite its large sample size. Second, we failed to systematically collect and quantify the frequency and quality of posture management implemented at home, such as time spent in the prone position daily. These behavioral factors significantly influence recovery and may serve as a key reason for the observed differences in recovery time within the group. Future studies should employ prospective designs, incorporating parental diaries or wearable devices for more precise evaluations of the role of environmental and behavioral interventions in spontaneous recovery. Additionally, the primary endpoint of this study was the normalization of cranial parameters, and long-term follow-up through early childhood was not conducted to assess the association between early cranial deformities and long-term neuropsychological development.

## 5 Conclusion

Multivariate analysis confirmed that age at diagnosis and severity of deformity were independent predictors of natural resolution of infantile positional cranial deformities. This finding reveals the existence of a critical recovery period during early infancy and demonstrates the necessity of implementing risk-stratified management. Future research should focus on integrating the aforementioned predictors into standardized clinical pathways and delving into the interactive effects of multiple factors, including genetic, environmental, and behavioral influences, to drive the transformation of clinical intervention strategies toward early prevention and precision interventions.

## Data Availability

The raw data supporting the conclusions of this article will be made available by the authors, without undue reservation.

## References

[1] Biggs WS. Diagnosis and management of positional head deformity. Am Fam Physician. (2003) 67(9):1953–6. 12751657

[2] Peitsch WK Keefer CH LaBrie RA Mulliken JB. Incidence of cranial asymmetry in healthy newborns. Pediatrics. (2002) 110(6):e72. 10.1542/peds.110.6.e72 12456939

[3] Linz C Kunz F Böhm H Schweitzer T. Positional skull deformities. Dtsch Arztebl Int. (2017) 114(31–32):535–42. 10.3238/arztebl.2017.0535 28835328 PMC5624275

[4] Park KM Tripathi NV Mufarrej FA. Quality of life in patients with craniosynostosis and deformational plagiocephaly: a systematic review. Int J Pediatr Otorhinolaryngol. (2021) 149:110873. 10.1016/j.ijporl.2021.110873 34380097

[5] Ifflaender S Rüdiger M Konstantelos D Lange U Burkhardt W. Individual course of cranial symmetry and proportion in preterm infants up to 6 months of corrected age. Early Hum Dev. (2014) 90(9):511–5. 10.1016/j.earlhumdev.2014.03.008 24751496

[6] Ifflaender S Rüdiger M Konstantelos D Wahls K Burkhardt W. Prevalence of head deformities in preterm infants at term equivalent age. Early Hum Dev. (2013) 89(12):1041–7. 10.1016/j.earlhumdev.2013.08.011 24016482

[7] Hussein MA Woo T Yun IS Park H Kim YO. Analysis of the correlation between deformational plagiocephaly and neurodevelopmental delay. J Plastic Reconstr Aesthet Surg. (2018) 71(1):112–7. 10.1016/j.bjps.2017.08.015 28958569

[8] Collett BR Kartin D Wallace ER Cunningham ML Speltz ML. Motor function in school-aged children with positional plagiocephaly or brachycephaly. Pediatr Phys Ther. (2020) 32(2):107–12. 10.1097/PEP.0000000000000687 32218071 PMC10507734

[9] Blanco-Diaz M Marcos-Alvarez M Escobio-Prieto I De la Fuente-Costa M Perez-Dominguez B Pinero-Pinto E. Effectiveness of conservative treatments in positional plagiocephaly in infants: a systematic review. Children. (2023) 10(7):1184. 10.3390/children10071184 37508680 PMC10378416

[10] Kang H. Importance of pediatrician’s role in preventing positional plagiocephaly. Clin Exp Pediatr. (2024) 67(6):294–5. 10.3345/cep.2023.01025 38772410 PMC11150983

[11] Solani B Talebian Ardestani M Boroumand H Ostadmohammadi V Hallajnejad M Kashani Zade M. Risk factors associated with positional plagiocephaly in healthy Iranian infants: a case-control study. Iran J Child Neurol. (2022) 16(2):85–92. 10.22037/ijcn.v16i1.28524 35497110 PMC9047834

[12] Watt A Alabdulkarim A Lee J Gilardino M. Practical review of the cost of diagnosis and management of positional plagiocephaly. Plast Reconstr Surg Glob Open. (2022) 10(5):e4328. 10.1097/GOX.0000000000004328 35702535 PMC9187200

[13] Pan W Liao J Tong X. Follow-up and prognostic study of infants with positional plagiocephaly. Zhongguo Dang Dai Er Ke Za Zhi. (2023) 25(4):368–73. 10.7499/j.issn.1008-8830.2210031 37073841 PMC10120334

[14] Glasgow TS Siddiqi F Hoff C Young PC. Deformational plagiocephaly: development of an objective measure and determination of its prevalence in primary care. J Craniofac Surg. (2007) 18(1):85–92. 10.1097/01.scs.0000244919.69264.bf 17251842

[15] Graham T Millay K Wang J Adams-Huet B O’Briant E Oldham M. Significant factors in cranial remolding orthotic treatment of asymmetrical brachycephaly. J Clin Med. (2020) 9(4):1027. 10.3390/jcm9041027 32260587 PMC7231243

[16] Clark TG Bradburn MJ Love SB Altman DG. Survival analysis part I: basic concepts and first analyses. Br J Cancer. (2003) 89(2):232–8. 10.1038/sj.bjc.6601118 12865907 PMC2394262

[17] Miyabayashi H Nagano N Kato R Noto T Hashimoto S Saito K. Cranial shape in infants aged one month can predict the severity of deformational plagiocephaly at the age of six months. J Clin Med. (2022) 11(7):1797. 10.3390/jcm11071797 35407405 PMC8999343

[18] Santander P Quast A Hubbert J Meyer-Marcotty P Hensel KO Bergmann C. The three-dimensional course of cranial development of very preterm infants during the first year of life. Early Hum Dev. (2024) 198:106131. 10.1016/j.earlhumdev.2024.106131 39427437

[19] Di Chiara A La Rosa E Ramieri V Vellone V Cascone P. Treatment of deformational plagiocephaly with physiotherapy. J Craniofac Surg. (2019) 30(7):2008–13. 10.1097/SCS.0000000000005665 31232996

[20] de Sousa YG Marques Lopes TL Soares ÁM Cavalcante MA Rangel JF de Macedo Filho L. Helmet versus non-helmet treatment in infants with positional cranial deformation: a systematic review and meta-analysis. J Clin Neurosci. (2025) 139:111431. 10.1016/j.jocn.2025.111431 40578012

[21] Binkiewicz-Glińska A Mianowska A Sokołów M Reńska A Ruckeman-Dziurdzińska K Bakuła S. Early diagnosis and treatment of children with skull deformations. The challenge of modern medicine. Dev Period Med. (2016) 20(4):289–95. 28216483

[22] Martín Molinari J Luján Molina G Muftoz-Serrano N. Positional plagiocephaly and neurodevelopment: a narrative review. Andes Pediatr. (2024) 95(5):620–8. 10.32641/andespediatr.v95i5.5150 39760633

[23] Pinyot J Lacambra D Garriga M Pinyot M Niubó JM. Positional plagiocephaly side and neurodevelopmental delay: study on 408 infants. J Craniofac Surg. (2024) 35(7):2027–35. 10.1097/SCS.0000000000010581 39418507

[24] Lidzba K Rodemann S Goelz R Krägeloh-Mann I Bevot A. Growth in very preterm children: head growth after discharge is the best independent predictor for cognitive outcome. Early Hum Dev. (2016) 103:183–8. 10.1016/j.earlhumdev.2016.09.016 27716567

[25] Nakanomori A Miyabayashi H Tanaka Y Maedomari T Mukai C Saito K. Changes in cranial shape and developmental quotient at 6 months of age in preterm infants. Children. (2023) 10(5):855. 10.3390/children10050855 37238403 PMC10217116

[26] Yang L Fu H Zhang L. A systematic review of improved positions and supporting devices for premature infants in the NICU. Heliyon. (2023) 9(3):e14388. 10.1016/j.heliyon.2023.e14388 36967878 PMC10031313

[27] Corte AD Rohde MA. Use of orthotic helmets in children with positional plagiocephaly and brachycephaly: a systematic review. Child’s Nerv Syst. (2025) 41(1):163. 10.1007/s00381-025-06826-0 40261429

