# Supplementary material for: Prognostic factors for cranial deformities in infancy: a retrospective cohort study
Source: Front Pediatr. 2026 Jun 25;14:1822648. doi: 10.3389/fped.2026.1822648 (PMC13345863; doi:10.3389/fped.2026.1822648)
Supplement: Supplementary file 1 [file Table1.docx]

**Supplementary Table 1. Baseline Characteristics of Preterm Infants**

| **Variables** | **Overall（n=228）** | **0-2months（n=118）** | **3-4months（n=101）** | **5-6months（n=9）** | **H/U** | **P** |
| --- | --- | --- | --- | --- | --- | --- |
| **Sex (n, %)** |  |  |  |  | 0.268 | 0.874 |
| Male | 117（51.32） | 62（52.54） | 51（50.50） | 4（44.44） |  |  |
| Female | 111（48.68） | 56（47.46） | 50（49.45） | 5（55.56） |  |  |
| **Gestational Age at Birth** (weeks, mean ± SD) | 32.43±3.90 | 31.78±6.66 | 33.17±4.30 | 32.00±3.14 | 13.085 | **0.001** |
| **Birth Weight** | 1870.79±644.25 | 1734.48±594.32 | 1995.54±588.03 | 2257.78±1284.61 | 10.476 | **0.003** |
| **Maternal Age** | 33.96±4.11 | 34.25±4.27 | 33.80±3.52 | 32.00±6.18 | 2.016 | 0.365 |
| **Cranial Morphology Type** |  |  |  |  | 5.741 | 0.453 |
| Plagiocephaly | 117（51.32） | 67（56.78） | 45（44.55） | 5（55.56） |  |  |
| Brachycephaly | 22（9.65） | 9（7.63） | 12（11.88） | 1（11.11） |  |  |
| Asymmetric Brachycephaly | 31（13.60） | 30（25.42） | 17（16.83） | 2（22.22） |  |  |
| Scaphocephaly | 58（25.43） | 12（10.17） | 27（26.74） | 1（11.11） |  |  |
| **Severity of Abnormality (n, %)** |  |  |  |  | 1.114 | 0.571 |
| Mild | 111（48.68） | 61（51.69） | 45（44.55） | 5（55.56） |  |  |
| Moderate | 72（31.58） | 34（28.81） | 35（34.65） | 3（33.33） |  |  |
| Severe | 45（17.74） | 23（19.50） | 21（20.80） | 1（11.11） |  |  |
