# Supplementary material for: Prognostic factors for cranial deformities in infancy: a retrospective cohort study
Source: Front Pediatr. 2026 Jun 25;14:1822648. doi: 10.3389/fped.2026.1822648 (PMC13345863; doi:10.3389/fped.2026.1822648)
Supplement: Supplementary file 2 [file Table2.docx]

**Supplementary Table 2. Baseline Characteristics of Term Infants**

| **Variables** | **Overall（n=991）** | **0-2months（n=52）** | **3-4months（n=770）** | **5-6months（n=169）** | **H/U** | **P** |
| --- | --- | --- | --- | --- | --- | --- |
| **Sex (n, %)** |  |  |  |  | 0.013 | 0.993 |
| Male | 519（52.37） | 27（51.92） | 404（52.47） | 88（52.07） |  |  |
| Female | 472（47.63） | 25（48.08） | 366（47.53） | 81（47.93） |  |  |
| **Gestational Age at Birth** (weeks, mean ± SD) | 38.85±3.37 | 39.02±2.69 | 38.84±3.36 | 38.82±3.57 | 1.172 | 0.557 |
| **Birth Weight** | 3264.45±496.57 | 3369.62±601.38 | 3365.09±484.00 | 3235.31±517.57 | 4.198 | 0.123 |
| **Maternal Age** | 33.73±10.15 | 32.88±3.53 | 33.87±11.36 | 33.33±3.45 | 0.706 | 0.703 |
| **Cranial Morphology Type** |  |  |  |  | 8.147 | 0.228 |
| Plagiocephaly | 590（59.54） | 29（55.77） | 468（60.78） | 93（55.03） |  |  |
| Brachycephaly | 330（33.30） | 0（0.00） | 244（31.69） | 5（2.96） |  |  |
| Asymmetric Brachycephaly | 42（4.24） | 0（0.00） | 34（4.42） | 8（4.73） |  |  |
| Scaphocephaly | 29（2.92） | 23（44.23） | 24（3.11） | 63（37.28） |  |  |
| **Severity of Abnormality (n, %)** |  |  |  |  | 1.984 | 0.371 |
| Mild | 773（78.00） | 40（76.93） | 608（78.96） | 125（73.96） |  |  |
| Moderate | 160（16.15） | 8（15.38） | 119（15.46） | 33（19.53） |  |  |
| Severe | 58（5.85） | 4（7.69） | 43（5.58） | 11（6.51） |  |  |
